# Catestatin-A Novel Predictor of Left Ventricular Remodeling After Acute Myocardial Infarction

**DOI:** 10.1038/srep44168

**Published:** 2017-04-11

**Authors:** Dan Zhu, Hong Xie, Xinyu Wang, Ying Liang, Haiyi Yu, Wei Gao

**Affiliations:** 1 Department of Cardiology, Peking University Third Hospital, Key Laboratory of Cardiovascular Molecular Biology and Regulatory Peptides, Ministry of Health (Peking University Third Hospital), Beijing, China

## Abstract

Catestatin was discovered as a potent inhibitor of catecholamine secretion and plays important roles in the cardiovascular system. Our previous study demonstrates a close relationship between catestatin levels and prognosis of ST-elevation myocardial infarction (STEMI). Using the same population, the goal of this study is to investigate the ability of catestatin to predict left ventricular (LV) remodeling in STEMI patients. 72 patients and 30 controls were included. Catestatin was sampled after admission to the emergency room (ER), at day3 (D3), and day7 (D7) after STEMI. Echocardiography was performed at D3 and after 65 months for evaluation of LVEDD, EF, IVS, LVPW, E, A, E’, E/A, and E/E’. The changes of these parameters from D3 to 65 months were used to reflect the changes of ventricular structure and function. We found that plasma catestatin levels at D3 were highly correlated with the changes of LVEDD, EF, E, A, E’, E/A, as well as E/E’. Patients with higher catestatin levels developed worse ventricular function during the follow-up period. Single-point catestatin was effective to predict LVEDD change. And concurrently increasing catestatin and NT-proBNP levels predicted the highest risk of LV remodeling. This study suggests an important prognostic information of catestatin on LV remodeling.

Left ventricular (LV) remodeling refers to the process by which ventricular size, structure and function are regulated by mechanical, neurohormonal, and genetic factors (refer to review^1^). It is driven by a combination of pathologic cardiomyocyte hypertrophy, cardiomyocyte apoptosis, myofibroblast proliferation, and interstitial fibrosis^2^. Following acute myocardial infarction (AMI), LV remodeling is a major predictor of morbidity and mortality^3^. Currently, echocardiography has been recommended as a routine examination to assess LV function in clinic^4^. However, it’s still not ubiquitously used because of high cost and need of specialists, especially in developing countries. In addition, when echocardiography shows positive signs, LV remodeling has already happened, which prevents its usage as an early indicator. Therefore, it’s essential to find a relatively easy and cost-effective way to identify patients with higher risk of LV remodeling at early stage after AMI, which promotes further interventions to prevent LV dilation.

In early studies, researchers demonstrated that the degree of chamber remodeling was highly associated with the extent of myocardial injury^3^ and factors like anterior infarct location^5^ and patency of the infarct-related artery^6^, etc. Pathologic LV remodeling is also closely linked to activation of a series of neuroendocrine, such as renin-angiotensin-aldosterone axis and the adrenergic nervous system. For clinical use, some biomarkers had been identified to predict LV remodeling, which include B-type natriuretic peptide (BNP)^7^, cardiac troponin I^8^, and serum Tenascin-C^9^. However, the predictive role of catestatin in LV remodeling has never been investigated.

Catestatin is a 21-amino acid peptide generated endogenously by proteolytic cleavage of its precursor chromogranin A (CHGA)^10^, which is co-stored and co-released with catecholamines. It has been intensively studied in cardiovascular system in recent years, which displayed potent vasodilatory effect in both rate and human^11^,^12^. In addition, catestatin exerts cardio-protective influence under Ischemia/Reperfusion condition, proved by several groups^13^,^14^. Our previous studies also indicated that catestatin had a predictive effect to detect stage B heart failure^15^ and was correlated to prognosis of patients with AMI^16^. Based on these functions in the cardiovascular system, we hypothesized that catestatin level might be useful for the diagnosis and determination of LV remodeling following AMI. So in this study, we assessed plasma catestatin concentrations with reference to LV function.

## Results

### Baseline characteristics

The baseline demographics, echocardiography and other laboratory findings are detailed in Table 1. In summary, 72 patients (age, 61.3 ± 14.4; sex, 81.9% male) with STEMI and 30 control subjects (age, 59.8 ± 10.4; sex, 76.7% male) were included. The baseline characteristics were compared between Control and STEMI patients. Patients with AMI had dramatically higher heart rates, Hs-CRP concentrations, as well as fasting glucose levels. For the echocardiography parameters, the EF, E, A, E’, E/E’ of AMI patients were significantly lower than those of control subjects, but other factors were comparable such as age, gender, etc.

### Correlation coefficients

**Figure 1:**
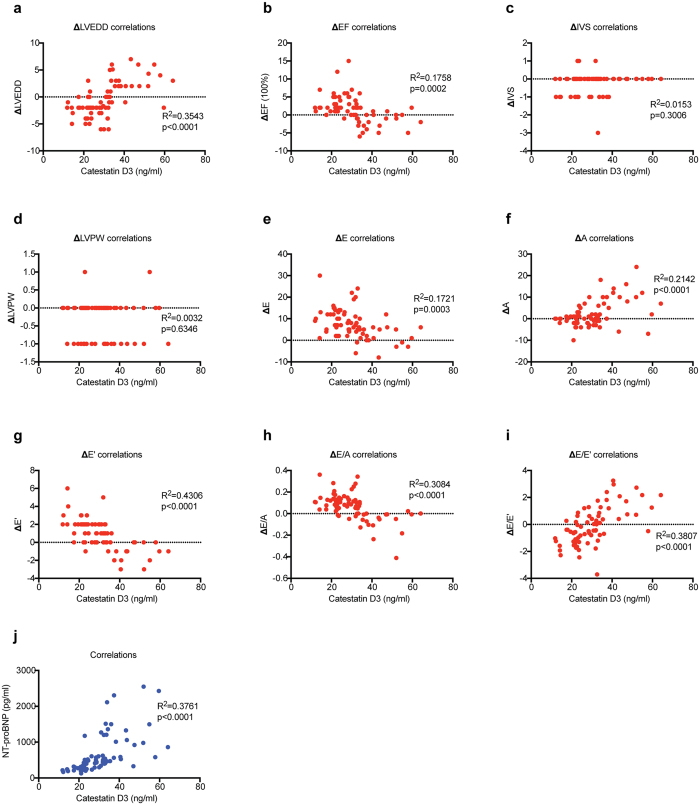
(**a–i**) Scatter plots (with Pearson or Spearman’s correlation coefficients) showing the correlation between catestatin concentrations at D3 and changes of echocardiography parameters including LVEDD, EF, IVS, LVPW, E, A, E’, E/A, and E/E’. j, Scatter plots showing the correlation between catestatin concentrations at D3 and NT-proBNP concentrations.

We have earlier reported that plasma catestatin achieved its highest level at D3 after STEMI (Supplementary Figure 1), which was an independent predictor of adverse events^16^. Here the correlations between catestatin concentrations at D3 and ΔLVEDD, ΔEF, ΔIVS, ΔLVPW, ΔE, ΔA, ΔE’, ΔE/A, ΔE/E’ were analyzed. As shown in Fig. 1a–i, the concentrations of catestatin were highly correlated with the changes of LVEDD (p < 0.0001), EF (p = 0.0002), E (p = 0.0003), A (p < 0.0001), E’ (p < 0.0001), E/A (p < 0.0001), as well as E/E’ (p < 0.0001). In addition, we examined the relationship between catestatin and N-terminal pro b-type natriuretic peptide (NT-proBNP), and found these two parameters were positively correlative with each other (Fig. 1j).

### Comparison of catestatin levels and changes of echocardiography parameters according to different grouping methods

**Figure 2:**
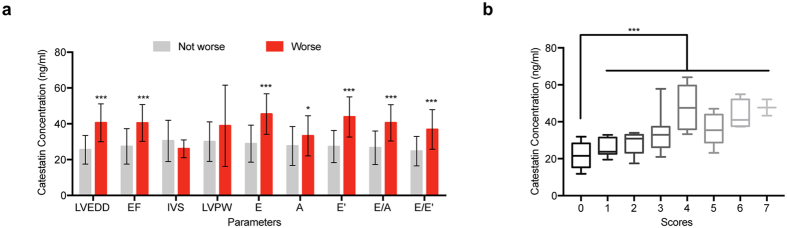
(**a**) Histograms showing the comparison of catestatin concentrations at D3 between “Worse” group and “Not worse” group. (**b**) Patients were stratified according to different scores, and catestatin concentrations were compared between these groups. Whiskers denote the minimal value and max value. Error bars, SD. *p < 0.05, **p < 0.01, ***p < 0.001.

First, patients were divided into 2 groups based on the median level of catestatin at D3 (28.71 ng/ml); the changes of echocardiography parameters as well as other characteristics listed in Table 1 were compared between these 2 groups. As summarized in Table 2, the high catestatin group had increased LVEDD, decreased EF, decreased E, increased A, decreased E’, decreased E/A, as well as increased E/E’, all of which are indicative to worse LV function, but there was no difference of other characteristics except for higher diastolic blood pressure and LDL cholesterol concentration in high catestatin group. When we compared the percentage of patients who developed worse conditions (increased LVEDD, decreased EF, decreased E, increased A, decreased E’, decreased E/A, and increased E/E’) between these 2 groups, we found more patients in the high catestatin group tended to have LV remodeling (Table 3). Second, on the basis of changes of echocardiography parameters, patients were then separated into 2 sets: one was named as “Worse” (with increased LVEDD, or decreased EF, or increased IVS, or increased LVPW, or decreased E, or increased A, or decreased E’, or decreased E/A or increased E/E’, respectively); the other one was called “not Worse” (otherwise), we found that among these parameters detected, except for IVS and LVPW, patients with worse conditions had higher levels of catestatin (Fig. 2a). At last, we scored individual patient according to these 9 echocardiography parameters assessed: each parameter is 1 point, and the final score of each patient represents how many parameters became worse during the follow-up period, indicating the severity of LV remodeling (for example, if a patient only has worse LVEDD and worse EF, the score would be 2). Using this stratification strategy, we found patients with more severe outcomes tended to have higher catestatin concentrations (Fig. 2b).

### ROC analysis of catestatin level for predicting LV remodeling

**Figure 3:**
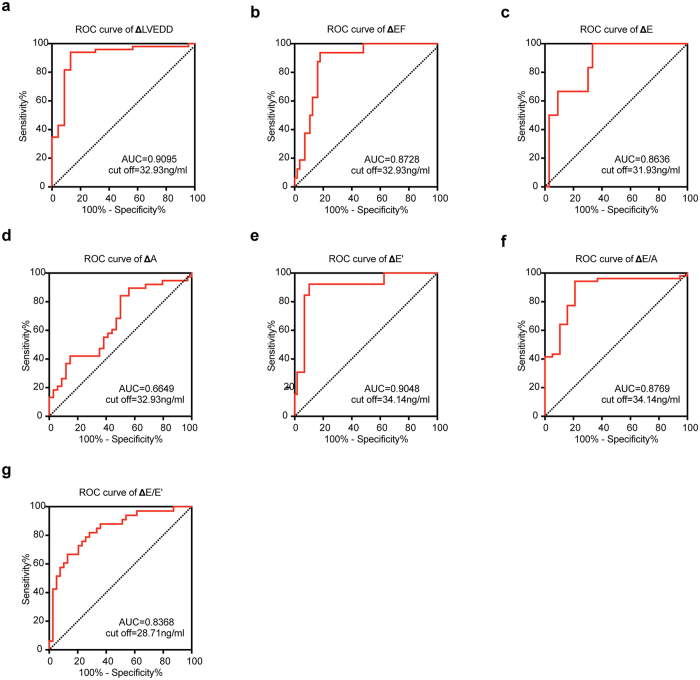
(**a–g**) Receiver operating characteristic curves (ROC) showing the ability of catestatin at D3 to predict echocardiography parameter changes. Specificity as well as sensitivity was determined. The diagonal was shown in each diagram.

We performed ROC analysis of catestatin levels at D3 for prediction of LV remodeling, as illustrated by the following echocardiography parameters: ΔLVEDD, ΔEF, ΔE, ΔA, ΔE’, ΔE/A, ΔE/E’ (Fig. 3a–g). The AUC for prediction of ΔLVEDD was 0.9095, the highest among the analyzed variables. The best cut-off value was 32.93 ng/ml, with a sensitivity of 93.88% and specificity of 86.96%. To compare the predicting value of catestatin and NT-proBNP, we also performed ROC analysis of NT-proBNP (Supplementary Figure 2), and we found that even though comparable, the AUCs of castestatin were slightly higher than that of NT-proBNP.

### Concurrently increasing catestatin and NT-proBNP levels predicted the highest risk of LV remodeling

Patients were grouped according to the catestatin and NT-proBNP median values (catestatin, 28.71 ng/ml; NT-proBNP, 472 pg/ml). Multivariate logistic regression found that concurrent increases in catestatin and NT-proBNP predicted the greatest risk [OR = 24.645 (95%CI: 2.869–211.698, p = 0.003)] (Table 4).

## Discussion

In STEMI, timely reperfusion therapy can reduce infarct size, preserve left ventricular function and improve clinical outcomes. However, as one of the strongest predictors of subsequent mortality, LV remodeling occurs in an appreciable proportion of patients with AMI, even those successfully treated with pPCI. Early identification of high-risk patients allows for early intervention with targeted treatment strategies and close follow-up. But currently, a major challenge in clinic is to discriminate between patients with reversible dysfunction and those who will experience little functional recovery accompanied by LV remodeling, which necessitates the development of new strategies to predict adverse events at early stages. It has been shown that the plasma concentration of catestatin is altered during the course of AMI, and it is associated with poor prognosis such as heart failure and death^16^. But the role of catestatin in the pathophysiological process of AMI and its relationship with LV remodeling are largely unknown. So in this study, we aim to assess the ability of catestatin level in plasma to predict functional recovery and extent of LV remodeling.

As a follow-up of our previous study^16^, the current study investigated the relationship between catestatin levels at D3 and the extent of LV remodeling, determined by the changes of LVEDD, EF, E, A, E’, E/A, and E/E’. We showed that catestatin was highly correlated with the changes of these parameters. In addition, when patients were separated into two groups according to the median concentration of catestatin, we found that patients with higher levels of catestatin tended to develop worse LV function. In addition, catestatin concentrations were also positively correlated with LV remodeling severity. At last, ROC analysis suggested catestatin as a prognostic indicator with high sensitivity and specificity. When comparing prognostic value of catestatin with well-known factor NT-proBNP, catestatin had slightly stronger effects in terms of ROC curves. And patients with concurrently increasing catestatin and NT-proBNP levels had the highest risk of worse LV functions. Overall, We demonstrated that a single measurement of catestatin, obtained at D3 after interventional management, provided useful prognostic information on changes in LV volume and function. Since catestatin can be measured in plasma collected with standard equipment, stored for prolonged period, and analyzed without tedious extraction procedures, developing the clinical use of catestatin might be of great importance.

Cardiac remodeling is generally accepted as a determinant of the clinical course of heart failure (HF), which can be considered a primary target for treatment and a reliable surrogate for long-time outcomes. It is manifested clinically as changes in structure and function of the heart resulting from cardiac load or injury, such as AMI. Using noninvasive imaging like echocardiography, physicians and researchers are able to analyze the mechanisms by which biochemical and cellular events contribute to these changes during remodeling. In the current study, the myocardial functions can be assessed through the following parameters: EF for systolic function; E, A, E’, E/A, and E/E’ for diastolic function. We found that patients with higher catestatin levels at D3 developed worse systolic as well as diastolic function during the follow-up period. It has been shown that in isolated avascular frog heart, catestatin dose-dependently decreased stroke volume and stroke work, noncompetitively inhibited the positive inotropic action of isoproterenol and the positive inotropic effect of endothlin-1^17^, suggesting its cardio-suppressive function. In addition, another group demonstrated the cardio-protective role of catestatin, which appeared mainly due to a direct reduction of post-ischemic myocardial damages and dysfunction, with infarct size reduced and left ventricular function improved^18^. Recently, Bassino *et al*. confirmed this effect using isolated adult rat cardiomyocytes undergoing simulated ischemia/reperfusion injury, and determined PI3K/NO/cGMP pathway as well as preservation of mitochondrial membrane potential were involved^19^. Because infarction expansion and post-ischemic reperfusion are two major factors contributing to myocardial function after AMI, it’s rationale to surmise that catestatin performed a protective role in this process.

Apart from the worsening of myocardial functions, our results also showed that catestatin levels at D3 were positively associated with the increase of LVEDD, a parameter indicating cardiac structure change. Because cardiomyocytes and extracellular matrix (ECM) are two essential components of the myocardium, the pathological alterations of them will provide insights into the remodeling process. For cardiomyocytes, the most important change after AMI is hypertrophy. Cardiomyocyte hypertrophy is characterized by an increase in cell size, enhanced protein synthesis, and heightened organization of the sarcomere^20^. It is a progressive response to the elevated load on the heart, which results in an increase of cardiac mass. It has been demonstrated for decades that the renin-angiotensis-aldosterone system (RAAS), regulated by catecholamine, contributes to the pathogenesis of cardiac hypertrophy^21^. As a peptide widely known to inhibit catecholamine release from both chromaffin cells and noradrenergic neurons^10^, catestatin performed a possible antihypertrophy function in cardiac tissue through the RAAS pathway. In addition, catestatin can lower the blood pressure through inducing histamine release from mast cells^22^, antagonizing both central neuronal nicotinic acetylcholine receptor (nAChR) and β-adrenoceptors (β-ARs) to decrease the sympathetic tone^23^, as well as decreasing reactive oxygen species (ROS) and increasing nitric oxide (NO). All of these suggested that catestatin exerts a protective action on hypertrophy by decreasing the pressure signal and cardiac afterload. For ECM, one of the most important pathological features of cardiac remodeling is fibrosis, and ECM degradation is closely involved in this process. Previous study showed that angiotensin II (Ang II) induced profibrogenic effects in vascular diseases^24^,^25^ and increased the expression and activity of various matrix metalloproteinase (MMPs), such as MMP-2 and MMP-9^25^. So the inhibition of catestatin on Ang II function performed an antifibrosis role of this peptide. Furthermore, sharing several common regulating factors with hypertrophy, cardiac fibrosis is also affected by catestatin through regulating NO and ROS production^26^,^27^. Taken together, catestatin participates in the regulation of related pathways and contributes to the inhibition of cardiac remodeling.

Petersen *et al*. demonstrated that catecholamine activation in patients with AMI, as measured by plasma adrenaline and noradrenaline, showed an early and rapid increase in the first 3 days and was restricted to the first 5 days, with values unchanged after 1 year compared to those at discharge, at day 6^28^. This study also suggested that the magnitude of the early catecholaminergic activation negatively correlated with left ventricular systolic performance^28^. As the inhibitor of catecholamine release, we speculated that the increased sympathoadrenal activities might promote negative feedback and increase the concentration of catestatin, which helps to alleviate the harmful effect of catecholamine. As mentioned before, catestatin exerts cardio-protective function through several mechanisms. Therefore, the increase of catestatin in patients with AMI may protect the myocardium. Because catestatin peaked within one week after infarction, and is relatively easy as well as cost-effective to measure, we suggest that it could be a useful and early predictive marker for LV remodeling. Furthermore, it’s not surprising that catestatin and NT-proBNP manifested a synergistic role in the prediction of LV remodeling after AMI, because NT-proBNP are substances that are produced in the heart and released in response to ventricular wall stress caused by volume and pressure overload, and it’s an established marker to evaluate the severity of heart failure. Therefore, tailoring the multifactor stratification strategy might largely increase the predictive values.

To the best of our knowledge, this is the first study presenting that catestatin might be a novel marker reflecting LV remodeling in the myocardium following AMI. In summary, we observed a positive association between catestatin concentrations at D3 and LV remodeling. However, despite the findings, there are still some limitations in the current study. First, the sample size was relative small. Second, this was a one-center study. Third, we just assessed the changes of echocardiographic parameters instead of giving a certain definition of LV remodeling, which might cause obscuration when making clinical decisions. Therefore, further large-scale investigations and careful comparisons are required to confirm the predictive ability of catestatin in LV remodeling in AMI patients.

## Methods

### Study population

A total of 117 consecutive patients with the first AMI admitted to the Department of Cardiology, Peking University Third Hospital, from June 2008 to April 2009 were included, 17 of them were lost to follow-up. In the rest of the 100 patients, we were able to get echocardiography parameters at D3 and 65 months from 72 of them, so this study is based on the information of these 72 patients. All patients received successful primary percutaneous coronary intervention (pPCI) within 12 h from the AMI symptoms onset. The ST segment elevation myocardial infarction (STEMI) was diagnosed according to the American College of Cardiology/American Heart Association guideline in 2004^29^. The study excluded patients with chronic obstructive pulmonary disease, significant kidney or hepatic diseases, tumor, and infectious diseases. During the same study period, 30 subjects who were admitted to the same hospital because of atypical chest pain but with normal coronary arteries confirmed by coronary angiography were included as controls, Resting blood pressure and heart rates (HRs) were measured at the same time when blood samplings were performed in triplicate in the supine position, using an oscillometric device (Philips IntelliVue MP20, Germany). This study was approved by the ethics review boards of Peking University Health Science Center. Written informed consent was obtained from the study population. All methods were performed in accordance with relevant guidelines and regulations.

### Blood Sampling

At the acute phase of AMI, blood samples were obtained from an antecubital vein without stasis in all patients immediately after admission to the emergency room (ER), and in the morning of the third day (D3) and the seventh day (D7) after AMI (n = 72). In the control subjects, blood samples were obtained from the antecubital vein in the morning of the same day when angiography was performed (n = 30). The blood samples, anticoagulated with ethylenediaminetetraacetic acid (EDTA), were immediately centrifuged at 3000 rpm for 10 min at 4 °C. An aliquot of the EDTA plasma was stored at −80 °C till analysis. Repeated freeze-thaw cycles were avoided.

### Assays

Plasma levels of catestatin were measured by enzyme-linked immunosorbent assay (ELISA) according to the manufacturer’s instruction (Cat. # EK-053-29, ELISA kit, Phoenix Pharmaceutical Inc., Burlingame, CA). The minimal detection limits for catestatin was 0.06 ng/ml. Serum NT-proBNP levels were measured by a two-site sandwich electrochemiluminescence immunoassay (Elecsys proBNP II, Roche Diagnostic, Mannheim, Germany). These assays were performed by an investigator blinded to the sources of the samples.

### Echocardiography Procedure

All patients underwent an echocardiography examination at D3 and 65 months after AMI in our hospital. A VIVID 7 (Vingmed, GE, Horten, Norway) scanner with a 3.3-MHz multiphase array probe was used for the Doppler echocardiography studies, which was conducted under the supervision of experienced cardiologists. The study commenced with each patient lying in the left decubitus position for probe insertion. The echocardiographic techniques were performed and cardiac dimensions and volumes calculated in accordance with the guidelines of the American Society of Echocardiography^30^. Transmitral pulsed Doppler was recorded in the apical 4-chamber view: early (E) and atrial (A) peak velocities (m/s), peak velocity E/A ratio, and E velocity deceleration time (ms) were obtained. Pulse-wave tissue Doppler imaging was performed using the Doppler tissue imaging function of the instrument. Sample volume was located at the lateral side of the mitral annulus. Early (E’) mitral annulus velocities and the E/E’ ratio were obtained.

### Statistics

Outcome measures were left ventricular end-diastolic dimension (LVEDD), ejection fraction (EF), interventricular septal end diastolic dimension (IVS), left ventricular posterior wall thickness (LVPW), E, A, E’, E/A, E/E’, and changes of them from D3 to 65 months (e.g. ΔLVEDD = LVEDD^65 months^-LVEDD^D3^). All results are expressed as the mean ± SD. Differences between groups were assessed by Student’s *t*-test and chi-square test. A Pearson or Spearman correlation was used to explore associations between catestatin levels and parameters. The sensitivity and specificity of catestatin concentration for predicting outcomes were determined, and receiver operating characteristics (ROC) curves were constructed. Univariate and multivariate logistic regression were performed to examine predictors of LV remodeling. p < 0.05 was considered statistically significant.

## Additional Information

**How to cite this article:** Zhu, D. *et al*. Catestatin-A Novel Predictor of Left Ventricular Remodeling After Acute Myocardial Infarction. *Sci. Rep.* **7**, 44168; doi: 10.1038/srep44168 (2017).

**Publisher's note:** Springer Nature remains neutral with regard to jurisdictional claims in published maps and institutional affiliations.

## Supplementary Material

Supplementary Information

## Figures and Tables

**Table 1:** Patient characteristics, echocardiography parameters and laboratory findings at baseline.

Variables	Control n = 30	STEMI n = 72	p value
Age (years)	59.8 ± 10.4	61.3 ± 14.4	0.6052
Male (%)	76.7	81.9	0.5407
Systolic blood pressure (mmHg)	129.8 ± 13.6	133.9 ± 17.5	0.2603
Diastolic blood pressure (mmHg)	75.0 ± 7.4	77.0 ± 13.2	0.4269
Heart rates (beats/min)	70.4 ± 8.8	82.4 ± 12.5	<0.0001
Diabetes mellitus (%)	23.3	22.2	0.9026
Hypertension (%)	70.0	63.9	0.5536
Hypercholesterolemia (%)	50.0	59.7	0.3663
Active smoker (%)	36.7	54.2	0.1072
β-blocker usage (%)	10.0	6.9	0.6010
Hs-CRP (mg/l)	1.68 ± 2.09	19.15 ± 23.45	0.0002
Creatinine (μmol/l)	101.4 ± 23.3	95.9 ± 16.1	0.1704
Fasting glucose (mmol/l)	5.1 ± 1.7	6.6 ± 2.4	0.0031
Total cholesterol (mmol/l)	4.7 ± 1.1	4.8 ± 1.1	0.7982
LDL cholesterol (mmol/l)	3.1 ± 1.0	3.1 ± 0.8	0.8215
LVEDD	50.0 ± 3.6	49.7 ± 4.9	0.7605
EF (%)	69.8 ± 4.8	52.6 ± 10.0	<0.0001
IVS	9.8 ± 0.7	9.7 ± 1.2	0.6085
LVPW	9.6 ± 0.7	9.7 ± 1.1	0.6349
E	87.8 ± 7.2	81.0 ± 10.5	0.0018
A	89.3 ± 10.6	82.6 ± 9.9	0.0041
E'	10.2 ± 2.3	8.7 ± 2.0	0.0011
E/A	1.0 ± 0.2	1.0 ± 0.2	0.9431
E/E'	8.8 ± 1.3	9.5 ± 1.2	0.0134

Values represent mean ± SD or the percentage of patients.

Hs-CRP, high-sensitivity C-reactive protein; LDL, low-density lipoprotein. LVEDD, left ventricular end-diastolic dimension; EF, ejection fraction; IVS, Interventricular septal end diastolic dimension; LVPW, left ventricular posterior wall thickness; E, peak early diastolic mitral flow velocity; A, peak late diastolic mitral flow velocity; E’, doppler-derived peak early diastolic mitral flow velocity.

**Table 2:** Comparison of changes of echocardiography parameters and other characteristics according to catestatin concentrations at D3.

Parameters	Low catestatin (<28.71 ng/ml)	High catestatin (>28.71 ng/ml)	p value
ΔLVEDD	−2.28 ± 1.78	1.05 ± 3.38	<0.0001
ΔEF	+3.25 ± 3.22	−0.08 ± 3.13	<0.0001
ΔIVS	−0.19 ± 0.53	−0.22 ± 0.64	0.8406
ΔLVPW	−0.22 ± 0.49	−0.25 ± 0.50	0.8115
ΔE	9.19 ± 5.72	5.44 ± 7.18	0.0167
ΔA	−0.25 ± 3.16	4.47 ± 7.14	0.0005
ΔE'	1.72 ± 1.30	0.08 ± 1.63	<0.0001
ΔE/A	0.11 ± 0.07	0.01 ± 0.14	0.0003
ΔE/E'	−0.76 ± 0.92	0.59 ± 1.43	<0.0001
Age (years)	60.9 ± 14.9	61.8 ± 14.1	0.7954
Male (%)	77.8	86.1	0.3580
Systolic blood pressure (mmHg)	135.1 ± 18.4	132.7 ± 16.9	0.5528
Diastolic blood pressure (mmHg)	73.6 ± 12.3	80.5 ± 13.2	0.0245
Heart rates (beats/min)	82.4 ± 13.2	82.3 ± 12.1	0.9629
Diabetes mellitus (%)	25.0	19.4	0.5708
Hypertension (%)	61.1	66.7	0.6236
Hypercholesterolemia (%)	66.7	52.8	0.2296
Active smoker (%)	50.0	58.3	0.4780
β-blocker usage (%)	5.6	8.3	0.6429
Hs-CRP (mg/l)	22.17 ± 26.14	16.13 ± 20.34	0.2779
Creatinine (μmol/l)	95.9 ± 18.8	95.8 ± 13.0	0.9711
Fasting glucose (mmol/l)	6.7 ± 2.3	6.5 ± 2.6	0.7350
Total cholesterol (mmol/l)	4.6 ± 1.0	5.0 ± 1.2	0.0923
LDL cholesterol (mmol/l)	2.9 ± 0.7	3.3 ± 0.8	0.0421

LVEDD, left ventricular end-diastolic dimension; EF, ejection fraction; IVS, Interventricular septal end diastolic dimension; LVPW, left ventricular posterior wall thickness; E, peak early diastolic mitral flow velocity; A, peak late diastolic mitral flow velocity; E’, doppler-derived peak early diastolic mitral flow velocity; Hs-CRP, high-sensitivity C-reactive protein; LDL, low-density lipoprotein.

**Table 3:** Percentage of patients who developed worse cardiac remodeling according to the catestatin concentrations at D3.

Parameters	Low catestatin (<28.71 ng/ml)	High catestatin (>28.71 ng/ml)	p value
LVEDD, n (%)	2 (5.6)	21 (58.3)	<0.0001
EF, n (%)	1 (2.8)	15 (41.7)	<0.0001
IVS, n (%)	2 (5.6)	1 (2.8)	0.5553
LVPW, n (%)	1 (2.8)	1 (2.8)	1.0000
E, n (%)	0 (0.0)	6 (16.7)	0.0105
A, n (%)	14 (38.9)	20 (55.6)	0.1567
E’, n (%)	1 (2.8)	12 (33.3)	0.0007
E/A, n (%)	2 (5.6)	17 (47.2)	<0.0001
E/E’, n (%)	7 (19.4)	26 (72.2)	<0.0001

LVEDD, left ventricular end-diastolic dimension; EF, ejection fraction; IVS, Interventricular septal end diastolic dimension; LVPW, left ventricular posterior wall thickness; E, peak early diastolic mitral flow velocity; A, peak late diastolic mitral flow velocity; E’, doppler-derived peak early diastolic mitral flow velocity.

The following conditions were considered to be worse: increased LVEDD; decreased EF; increased IVS; increased LPVW; decreased E; increased A; decreased E’; decreased E/A; or increased E/E’.

**Table 4:** Univariate and multivariate ORs for severity of LV remodeling.

Characteristics	Outcome		Univariate		Multivariate	
	Not wourse	Worse	OR (95% CI)	*P* value	OR (95% CI)	*P* value
Age (year) per 1 year			0.998 (0.963–1.035)	0.928	1.007 (0.957–1.058)	0.796
Sex						
Male	15	44	1		1	
Female	5	8	0.545 (0.154–1.926)	0.346	0.647 (0.118–3.540)	0.615
β-blocker usage						
No	19	48	1		1	
Yes	1	4	1.583 (0.166–15.094)	0.690	0.697 (0.026–18.520)	0.829
Diabetes mellitus						
No	15	41	1		1	
Yes	5	11	0.805 (0.240–2.703)	0.725	0.262 (0.033–2.094)	0.206
Hypertension						
No	8	18	1		1	
Yes	12	34	1.259 (0.436–3.640)	0.670	1.162 (0.175–7.694)	0.876
Combined						
0	15	12	1		1	
1	3	15	6.250 (1.461–26.739)	0.013	9.973 (1.371–72.559)	0.023
2	2	25	15.625 (3.067–79.594)	0.001	24.645 (2.869–211.698)	0.003
Hs-CRP per 1			0.994 (0.973–1.015)	0.570	0.998 (0.969–1.028)	0.898
Creatinine per 1			0.976 (0.945–1.008)	0.137	0.990 (0.949–1.033)	0.643
LVEDD per 1			0.998 (0.898–1.108)	0.963	0.925 (0.784–1.091)	0.354
EF per 1			0.948 (0.898–1.002)	0.059	0.908 (0.828–0.996)	0.041

Combined 1: (Catestatin>28.71 ng/ml & NT-proBNP<472 pg/ml or Catestatin<28.71 ng/ml & NT-proBNP>472 pg/ml) vs. (Catestatin<28.71 ng/ml & NT-proBNP<472 pg/ml); Combined 2: (Catestatin>28.71 ng/ml & NT-proBNP>472 pg/ml) vs. (Catestatin<28.71 ng/ml & NT-proBNP<472 pg/ml). Hs-CRP, high-sensitivity C-reactive protein; LVEDD, left ventricular end-diastolic dimension; EF; ejection fraction.
