# Supplementary material for: Catestatin-A Novel Predictor of Left Ventricular Remodeling After Acute Myocardial Infarction
Source: Sci Rep. 2017 Apr 11;7:44168. doi: 10.1038/srep44168 (PMC5387721; doi:10.1038/srep44168)

Supplementary Information

Catestatin-A Novel Predictor of Left Ventricular Remodeling After Acute Myocardial Infarction

Dan Zhu[1,3], Hong Xie[1,2,3] , Xinyu Wang[1], Ying Liang[1], Haiyi Yu[1], Wei Gao[1*]

Supplementary Figure 1 Histograms showing the distribution of plasma catestatin concentrations at different time points (ER, D3, and D7). The p values for the comparison are indicated.

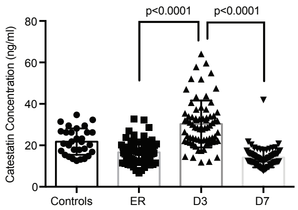

Supplementary Figure 2 Receiver operating characteristic curves (ROC) showing the ability of NT-proBNP to predict echocardiography parameter changes. Specificity as well as sensitivity was determined. The diagonal was shown in each diagram.

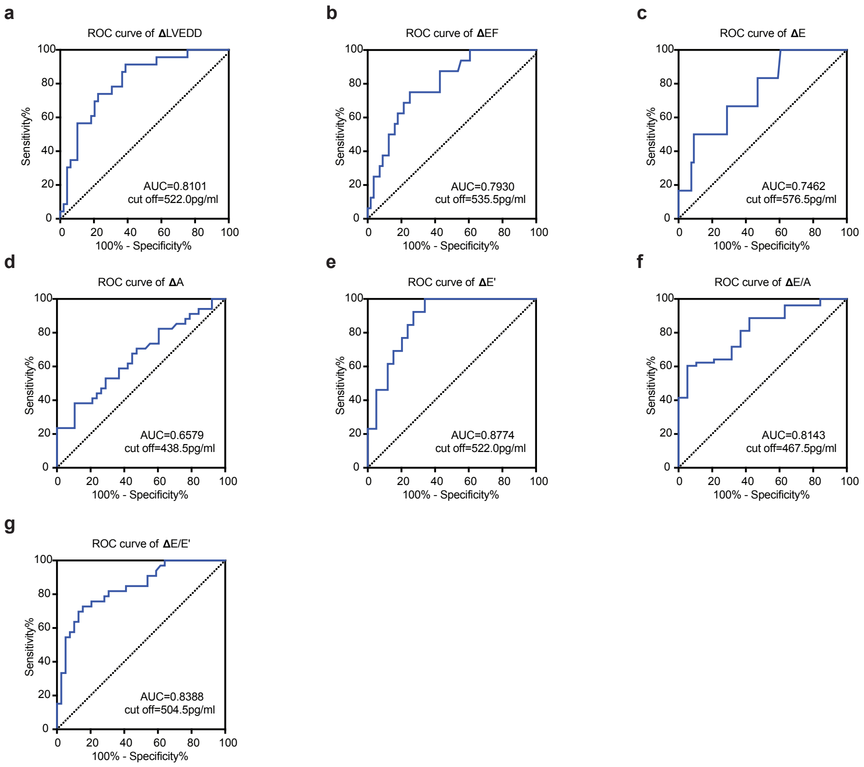

Supplement: Supplementary Information [file srep44168-s1.pdf]
